# Ergonomic benefit using heads-up display compared to conventional surgical microscope in Japanese ophthalmologists

**DOI:** 10.1371/journal.pone.0297461

**Published:** 2024-05-22

**Authors:** Motohiro Kamei, Hisaharu Suzuki, Hideyuki Terayama, Rana Ghafouri, Margaret H. Ainslie-Garcia, Nicole C. Ferko, Hang Cheng, Derek O’Boyle, Makoto Nakamura

**Affiliations:** 1 Department of Ophthalmology, Aichi Medical University, Nagakute, Aichi, Japan; 2 Zengyo Suzuki Eye Clinic, Fujisawa-shi, Kanagawa, Japan; 3 Alcon Japan Ltd., Tokyo, Japan; 4 Eversana Life Science Services, Burlington, Ontario, Canada; 5 Alcon Vision LLC, Fort Worth, Texas, United States of America; 6 Department of Ophthalmology, Kobe University, Kobe City, Hyogo, Japan; Jordan University of Science and Technology Faculty of Applied Medical Science, JORDAN

## Abstract

**Purpose:**

Occupational musculoskeletal disorders are prevalent in ophthalmic surgeons and can impact surgeons’ well-being and productivity. Heads-up displays may reduce ergonomic stress compared to conventional microscopes. This cross-sectional, non-interventional study compared ergonomic experience between heads-up display and conventional ocular microscopes.

**Methods:**

The study protocol was approved by the independent ethics committee and nonprofit organization MINS Institutional Review Board. An online questionnaire was distributed to a sample of ophthalmic surgeons in Japan with experience operating with heads-up display. The questionnaire captured surgeon-specific variables, the standardized Nordic Musculoskeletal Questionnaire, and custom questions to compare heads-up display and conventional microscope and understand long-term impacts of musculoskeletal disorders.

**Results:**

Analysis was conducted on responses from 67 surgeons with a mean 25 years of practice and 2.7 years using heads-up display. Many surgeons agreed or strongly agreed that heads-up display reduced the severity (40%) and frequency (40%) of pain and discomfort, improved posture (61%), and improved overall comfort (61%). Of respondents who experienced asthenopia (*n* = 59) or pain/discomfort during operation (*n* = 61), 54% reported improvement in asthenopia and 72% reported feeling less pain/discomfort since using heads-up display. Overall, 69% reported preference for heads-up display.

**Conclusion:**

This study provides novel data on musculoskeletal disorders and the long-term impacts of ergonomic strain reported by ophthalmologists building on existing literature demonstrating ergonomic and other advantages of heads-up display. Future studies with objective ergonomic assessment are warranted to validate these findings.

## Introduction

Occupational musculoskeletal disorders (MSDs) are prevalent among surgeons and have been shown to be the number one cause of absenteeism for health-care workers [1]. In particular, ophthalmologists have been established as a particularly high-risk group for MSDs [2–5]. Ophthalmic surgeries involve a combination of task repetition, cramped positions, and sustained non-neutral postures, which may contribute to the development of MSDs. A driving factor of these issues in ophthalmologists is the surgical microscope, which may cause musculoskeletal pain and discomfort due to the forward posture and unbalanced position of the head, neck and arms [6]. Sustained forward head posture involves flexion of the lower cervical spine with associated scapular protraction and over long periods may affect soft tissues, muscles, bony structures, and neural elements in the body, leading to neck-related MSDs [7]. There is evidence indicating that surgeons have concerns of negative long-term impacts of MSDs, leading them to change their surgical or retirement plans [4, 8, 9]. Early implementation of good ergonomic practices in the operating room (OR) is critical to mitigate musculoskeletal pain for surgeon well-being [6], however, studies on ergonomic interventions in the OR are limited [1].

In recent years, surgery with a heads-up display (HUD) has become possible for ophthalmology through digital visualization systems that integrate with the microscope, displaying the operating area on a large screen. In addition to enabling collaboration and training in the OR [10], these systems allow ophthalmologists to have a more neutral posture while operating. Preliminary studies of HUD have reported ergonomic benefits including improved neck and back comfort, however, these studies were limited in their sample size (≤ 20 surgeons) and design, and tasks were assessed in controlled study environments which may not be reflective of the pressures of surgery [11–13]. In more recent research, questionnaire-based techniques have been used with larger sample sizes to assess ergonomics and benefits of HUD in ophthalmology [4, 5]. One study found that 66% of ophthalmologists experienced work-related pain, with a significant correlation between presence of MSDs and time spent performing surgery (*P* < 0.01) [4]. Another survey assessing HUD use found that for surgeons using HUD in >50% of their cases, the odds of reporting an improvement in pain in the OR were 5.12-times greater than for those using HUD in ≤50% of cases (*P* = 0.029) [5]. However, both of these surveys were administrated to surgeons located in the United States (US), and generalizability to other countries may be limited.

Several important factors distinguish Asian populations from those in North America that may affect the prevalence of MSDs, the use of HUD and potential ergonomic benefits. For instance, US surgeons are often specialized in either anterior- or posterior-segment surgeries [5], while in many Asian countries, including Japan, surgeons may have multiple specialties and combination surgeries are often performed. Additionally, there may exist significant physical differences, such as average height and weight, or cultural distinctions that may influence the uptake and use of surgical tools.

To date, evidence for the effects of surgical ergonomics and work-related MSDs in Eastern countries, including Japan, is limited [1, 14]. Although one recent study evaluating Japanese endoscopists revealed that the majority (79%) had experienced occupational related-MSDs in at least one anatomic location during the previous year, and 17% had missed work due to severe MSDs [15], there is no data available specific to Japanese ophthalmologists. The objective of the current study was to investigate the presence of MSDs and whether HUD provides ergonomic or other benefits compared to conventional ocular microscopes, as reported by a sample of ophthalmologists in Japan.

## Methods

### Ethical considerations

The protocol for this non-interventional observational study was prepared according to the Helsinki Declaration and Japanese research guidelines including the Ethical Guidelines for Medical and Biological Research Involving Human Subjects. The comprehensive study protocol was reviewed and approved by an independent ethics committee at the non-profit organization MINS Institutional Review Board in Japan on September 9, 2021 (Protocol number CTT834-H001). Participants were provided with an electronic consent form, which informed them of the nature and purpose of the study, methods, benefits and risks, handling and storage of personal information, IRB approval, and information about who to contact if they had questions or concerns. Physicians were also informed that participation was voluntary and were notified of their right to withdraw at any time. All participants provided written informed consent prior to administration of the questionnaire.

### Study population and recruitment

Ophthalmic surgeons practicing in Japan who had passed residency and had experience using both conventional ocular microscopes and HUD were eligible to participate in this cross-sectional, single-masked study (data analyst masked). Ophthalmologists who had previously given consent to be contacted for participation in a survey, were contacted via email to participate by a third-party research agency between the periods of October to November 2021. Participants were informed it was an industry sponsored study, but participant identity was masked from the Principal Investigators and the manufacturer. Participants were compensated with an honorarium upon completion or could opt to decline the honorarium. Only one author (RG) had access to information that could identify individual participants, for the purposes of managing recruitment. No personal identifying information was present in the data files used for analysis.

### Questionnaire

The questionnaire used by Weinstock et al. [5] was adapted for use in Japan, with guidance from the Principal Investigators and a literature review. In brief, the third-party research agency who developed the original questionnaire performed the adaptation for the Japan region under the guidance of native language speakers and the three Principal Investigators, experts in the field of surgical ophthalmology covering the therapeutic areas of retina, cataract, and glaucoma. All three Principal Investigators contributed to, reviewed, and approved the adaptation process. A native language speaker translated the questionnaire. The translation was independently verified by two additional native language speakers. Several questions were modified to be more culturally appropriate (eg, adding “acupuncture” to past medical treatments), including a question to understand breakdown of surgeries by type, as combination surgeries are often performed in Japan but not in the United States. Questions related to eye strain and the impact of musculoskeletal disorders on productivity were added to inform regional research questions of interest to the Principal Investigators. For the complete questionnaire, see the Supporting Information (S1 File). Part one of the questionnaire collected participant characteristics (e.g., age, height, specialty, years of practice), musculoskeletal health history (e.g., baseline musculoskeletal pain and headache, posture during surgery, prior injuries), surgical variables (e.g., case volume, average case length, teaching facility), and HUD use (e.g., years using HUD, proportion of surgeries performed with HUD). In part two, the standardized Nordic Musculoskeletal Questionnaire (NMQ) was used to assess pain-related trouble in participants, defined as ache, pain, discomfort, and/or numbness. Part three included questions to assess potential long-term impacts of MSDs, which were adapted from Schechet et al. [4]. Questions in part four compared surgeons’ perceptions of headache, pain, benefit, and preference between HUD and conventional ocular microscopes.

### Statistical analysis

Descriptive analyses were performed to summarize population characteristics and questionnaire responses for the complete sample. Descriptive analyses were also conducted with respondents divided into the following three subgroups, based on the most commonly performed procedures. Since all surgeons performed cataract surgery, the subgroups were defined as follows: 1) cataract-specialty surgery group (i.e., 80% or more were cataract-only surgeries, without combination procedures); for physicians whose surgeries consisted of less than 80% cataract-only surgeries, they were divided into, 2) anterior group (i.e., sum of anterior eye surgeries [glaucoma and corneal surgery] greater than the sum of posterior eye surgeries [retinal surgery]), and 3) posterior group (i.e., sum of posterior eye surgeries [retinal surgery] greater than the sum of anterior eye surgeries [glaucoma and corneal surgeries]).

To explore variables that may predict improvement in pain-related issues when performing surgery, a multivariable regression analysis was performed. Recommendations from Principal Investigators informed the variables to be tested: age, years of practicing ophthalmology, years of experience using HUD, proportion of surgeries with HUD use, practice at teaching facility, height, reported pain in the past 12 months (from the NMQ), and device preference. Interaction effects were tested between variables related by time (i.e., age, years practicing ophthalmology, years of experience using HUD). The model fit was addressed using the Akaike Information Criterion (AIC) and variable selection was performed using a stepwise selection methodology. A *P*-value < 0.05 was considered statistically significant. Statistical analysis was performed in R, version 3.6.1 (RStudio, Vienna, Austria).

## Results

### Surgeon characteristics

The questionnaire was completed by 67 surgeons, with a response rate of 94%. Of these, 60 (90%) identified as attending surgeons (i.e., after 6^th^ year as an ophthalmologist) and 4 (6%) identified as fellows (i.e., 4–5 years as an ophthalmologist). The remaining three (4%) identified as “Other”, with specifying “Private practice.” Respondents reported their surgical specialties, which were not mutually exclusive as Japanese ophthalmologists perform surgery for a wide range of ophthalmic diseases in addition to their area of specialty. Sixty-one (91%) respondents identified as cataract specialists, 54 (81%) were retinal specialists, 31 (46%) were glaucoma specialists, and 9 (13%) were corneal specialists. Population characteristics including OR variables and baseline pain variables are summarized in **Table 1**.

**Table 1:** Population characteristics.

Characteristic	Total
	(N = 67)
Male (*n*, %)	64 (95.5%)
Female (*n*, %)	3 (4.5%)
Height, cm (mean, SD)	171.76 (5.98)
Weight, kg (mean, SD)	71.37 (10.53)
Age (mean, SD)	51.15 (8.67)
Years practicing ophthalmology (mean, SD)	25.00 (8.86)
Years using HUD (mean, SD)	2.73 (1.69)
**Operating Room Variables**	
Operating position (*n*, %)	
Temporal in >90% of cases	6 (9.0%)
Superior in >90% of cases	52 (77.6%)
Mixed distribution	9 (13.4%)
Type of Microscope (*n*, %)	
Floor Mounted	54 (80.6%)
Ceiling Mounted	13 (19.4%)
Teaching facility, yes (*n*, %)	46 (68.7%)
Total time spent in surgery, per operating day, minutes (mean, SD)	276.67 (133.01)
Average time to complete most frequent surgical case, minutes (mean, SD)	32.22 (29.04)*
Estimated annual case volume (mean, SD)	730.87 (568.13)
Estimated proportion of surgeries performed with HUD (*n*, %)	
1%-25%	24 (35.8%)
26%-50%	12 (17.9%)
51%-75%	2 (3.0%)
76%-100%	29 (43.3%)
**Baseline Pain Characteristics**	
Headache severity, 0–10 (median, IQR)^†^	0.00 (0–2.00)
Severity for those experiencing headaches, 0–10 (median, IQR)^†^^,^ ^‡^	2.00 (1.00–3.50)
Severity of neck or back pain/discomfort, 0–10 (median, IQR)^§^	1.00 (0–4.00)
Nordic Musculoskeletal Questionnaire	
Neck (*n*, %)	32 (47.8%)
Upper Back (*n*, %)	21 (31.3%)
Lower Back (*n*, %)	35 (52.2%)
Shoulder (right) (*n*, %)	19 (28.4%)
Shoulder (left) (*n*, %)	21 (31.3%)
None of the above (*n*, %)	22 (32.8%)

* Value presented after removal of outliers (n = 3) who reported abnormally long durations for one procedure (12, 8, and 6 hours).

† Surgeons were asked to rank their average headache severity on a scale of 0–10 (0 = no headaches, 5 = moderate headaches, and 10 = worst possible headache).

‡ Surgeons who reported “0” for average headache severity (n = 35) were removed.

^§^ Surgeons were asked to rank their average level of neck or back pain/discomfort on a scale of 0–10 (0 = no pain, 5 = moderate pain, and 10 = worst possible pain).

**Abbreviations:** cm = centimeters; HUD = heads-up display; kg = kilograms; IQR = interquartile range; SD = standard deviation.

The majority of surgeons used the NGENUITY 3D visualization system (*n* = 62; 93%) followed by Zeiss ARTEVO 800 (4; 6%) and the Sony HD Medical Display system (1; 1%). The mean duration of HUD use was 2.7 years, and the mean proportion of surgeries performed with HUD was 54%.

### Ergonomics and reported benefits of HUD in the OR

**Fig 1:**
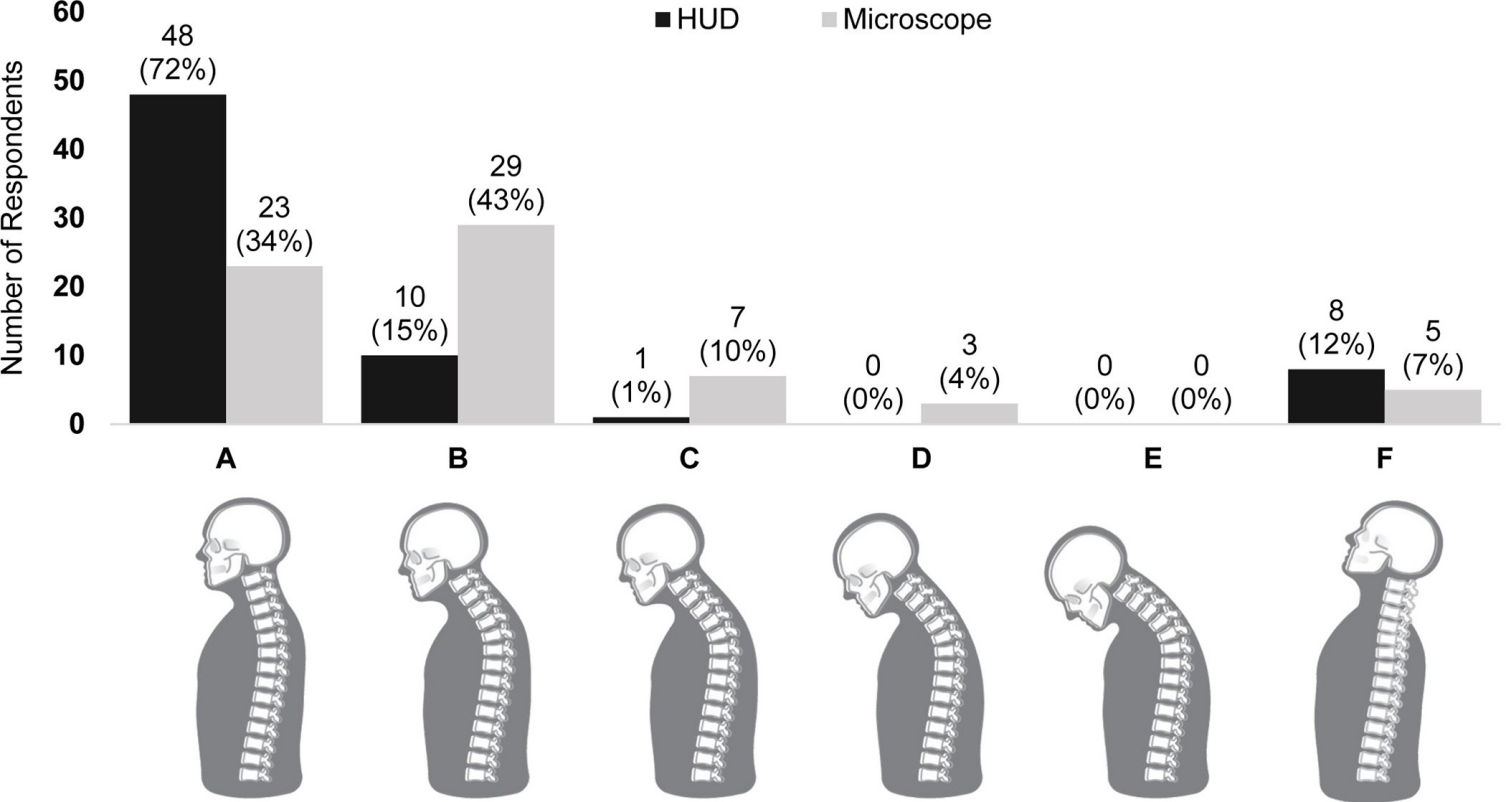
Reported posture when using heads-up display versus conventional microscope. **Abbreviations**: HUD = heads-up display.

**Fig 2:**
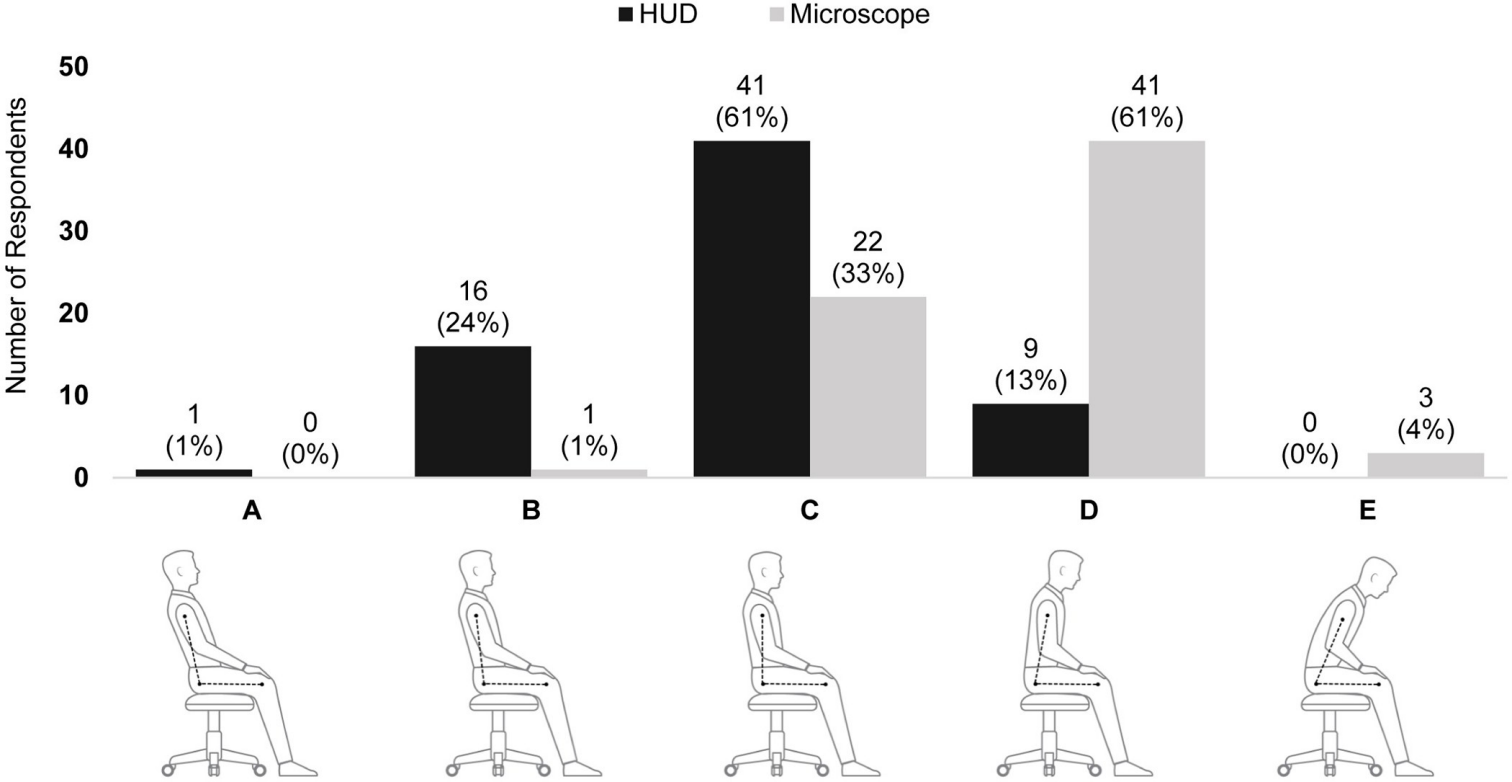
Reported angle of back when using heads-up display versus conventional microscope. **Abbreviations**: HUD = heads-up display.

When asked to identify their posture and angle of their back while using HUD and conventional microscope, 72% of surgeons reported a neutral neck posture while using HUD, versus only 34% when using a microscope (Panel A, **Fig 1**). Some degree of forward head posture was reported by 57% of surgeons when using a microscope, compared to only 16% when using HUD (Panels B-E, **Fig 1**). With respect to the reported back angle, 61% of surgeons reported a neutral back position while operating with HUD, versus only 33% when using a microscope (Panel C, **Fig 2**); in contrast, only 13% reported a forward bent position when using HUD versus 65% when using a microscope (Panels D-E, **Fig 2**).

**Fig 3:**
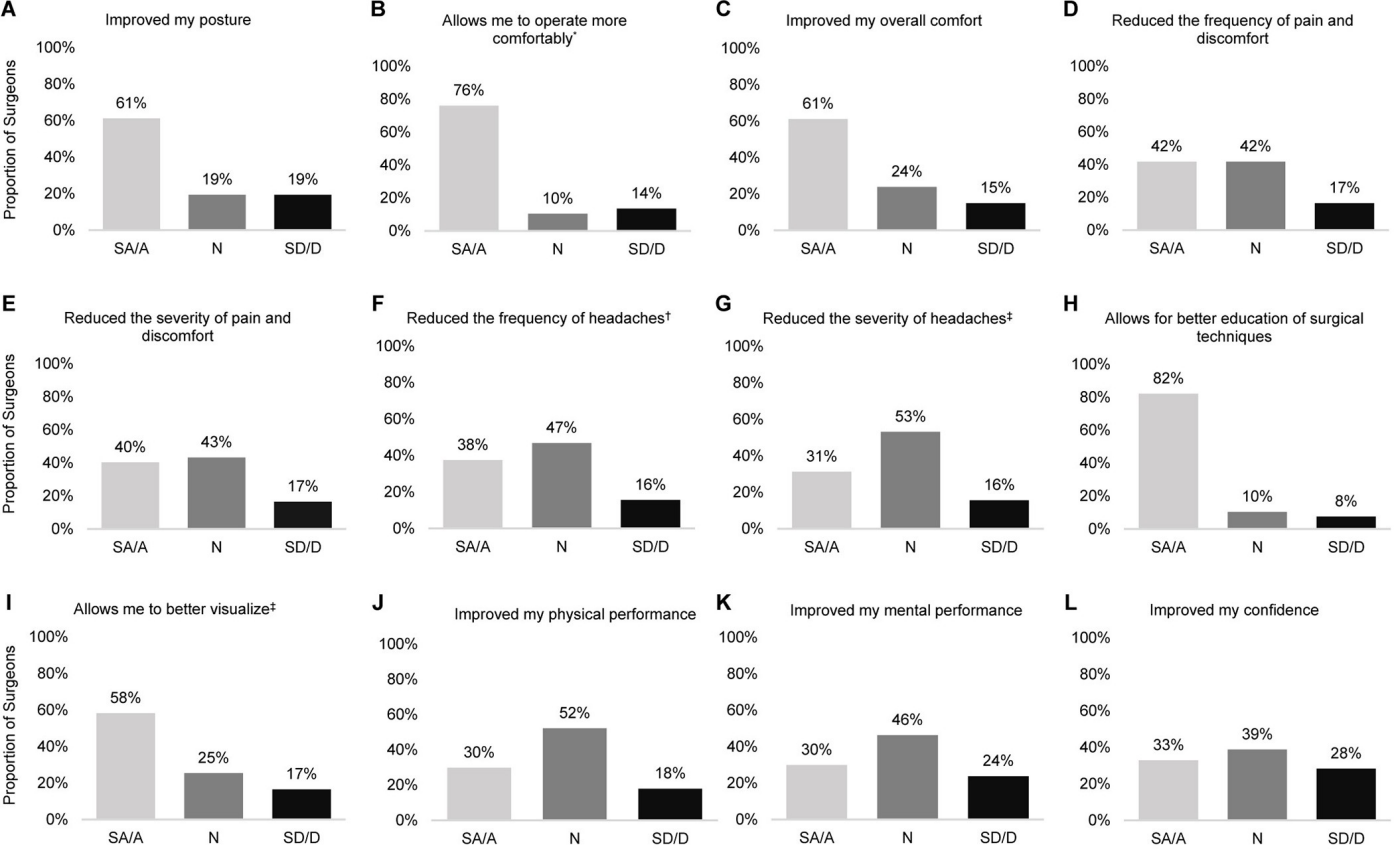
Frequency distribution for level of agreement to the statement: “Compared to a conventional microscope, the use of a heads-up visualization system in the operating room…”. * Allows me to operate more comfortably under higher magnification.† Respondents who selected a rating of “0 –No headaches” for average baseline headache severity (n = 35) were removed. ‡ Allows me to better visualize the areas and angles required for the procedure. **Abbreviations:** N = neutral; SA/A = strongly agree or agree; SD/D = strongly disagree or agree.

The majority of surgeons reported improvements in asthenopia (32/59; 54%), pain and/or discomfort during operation (44/61; 72%) and pain-related issues (34/54; 63%) in the OR since operating with HUD. Most surgeons strongly agreed or agreed (SA/A) that compared to a conventional microscope, HUD had improved posture (61%), allowed them to operate more comfortably under higher magnification (76%), and improved overall comfort (61%) (**Fig 3**). A considerable proportion also noted benefits for reduced frequency (42%) and severity (40%) of pain and discomfort, and reduced frequency (38%) of headaches for those reporting headaches at baseline; however, a lower proportion SA/A that HUD reduced headache severity (31%) (**Fig 3**). The use of HUD in the OR was also associated with several other benefits, including better education of surgical techniques (82%), and better visualization of the areas and angles needed for operation (58%). On the other hand, although identified as a benefit by roughly one third of participants, agreement on HUD supporting improved physical performance (defined as fatigue, stamina, and mobility) (30%), mental performance (defined as ability to focus and mental clarity) (30%), and confidence while operating (33%) was lower (**Fig 3**).

### Surgeon specialty subgroups

**Fig 4:**
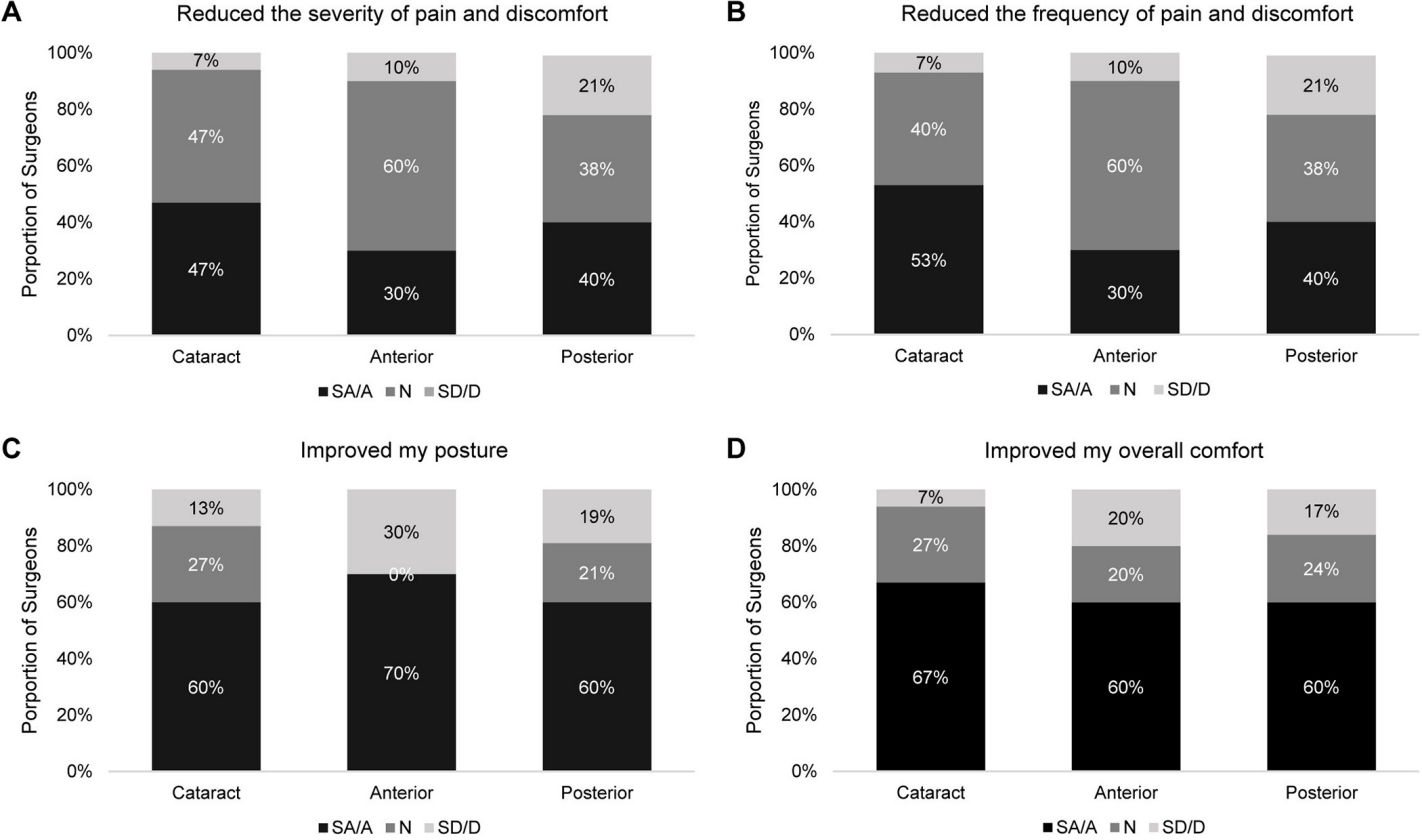
Distribution of perceived benefits of heads-up display by surgeon subgroup. **Abbreviations:** N = neutral; SA/A = strongly agree or agree; SD/D = strongly disagree or agree.

An exploratory subgroup analysis divided surgeons based on their most commonly performed procedures, to evaluate any variations in their characteristics or reported benefits of HUD (**Table 2**). Of the three subgroups, the greatest proportion of surgeons from the cataract-specialty surgery group reported a reduction in severity and frequency of pain and/or discomfort and improvements in overall comfort during surgery (**Fig 4**). For improvements in posture, a higher proportion of surgeons in the anterior surgery group agreed or strongly agreed that HUD improved their posture (70% [7/10]) versus 60% in the cataract or posterior surgery groups (**Fig 4**). Of note, 27% in the cataract and 21% in the posterior surgery group responded “Neutral” for improvements in posture, while none of the respondents in the anterior surgery group reported “Neutral”.

**Table 2:** Surgeon subgroup population characteristics.

	Cataract	Anterior	Posterior
	(*n* = 15)	(*n* = 10)	(*n* = 42)
Age (mean, SD)	54.27 (9.76)	50.60 (9.19)	50.17 (8.08)
Total time spent in surgery, per operating day, minutes (mean, SD)	181.13 (86.11)	342.00 (157.61)	295.24 (125.55)
Average time to complete most frequent surgical case, minutes (mean, SD)*	11.33 (4.56)	38.50 (24.04)	35.03 (30.84)
Estimated annual case volume (mean, SD)	855.33 (610.56)	576.00 (550.94)	723.29 (560.43)
Estimated proportion of surgeries performed with HUD (*n*, %)			
1%-25%	3 (20.0%)	7 (70.0%)	14 (33.3%)
26%-50%	2 (13.3%)	1 (10.0%)	9 (21.4%)
51%-75%	0 (0%)	0 (0%)	2 (4.8%)
76%-100%	10 (66.7%)	2 (20.0%)	17 (40.5%)
Headache severity, 0–10 (median, IQR)^†^	0.00 (0.00–0.50)	1.00 (0.00–2.00)	1.00 (0.00–2.00)
Severity for those experiencing headaches, 0–10 (median, IQR)^†^^,^ ^‡^	1.00 (1.00–2.25)	2.00 (1.25–4.25)	2.00 (1.00–3.00)
Neck and back pain severity, 0–10 (median, IQR)^§^	0.00 (0.00–1.50)	0.50 (0.00–1.00)	2.50 (0.00–5.00)
Nordic Musculoskeletal Questionnaire			
Neck (*n*, %)	4 (26.7%)	5 (50.0%)	23 (54.8%)
Upper Back (*n*, %)	2 (13.3%)	2 (20.0%)	17 (40.5%)
Lower Back (*n*, %)	7 (46.7%)	5 (50.0%)	23 (54.8%)
Shoulder (right) (*n*, %)	3 (20.0%)	2 (20.0%)	16 (38.1%)
Shoulder (left) (*n*, %)	3 (20.0%)	2 (20.0%)	14 (33.3%)
None of the above (*n*, %)	6 (40.0%)	3 (30.0%)	13 (31.0%)

* Value presented after removal of outliers who reported abnormally long durations for one procedure: two from the Posterior group (12 hours and 8 hours) and three from the Cataract-specialty group (6 hours, 100 minutes, and 40 minutes)

† Surgeons were asked to rank their average headache severity on a scale of 0–10 (0 = no headaches, 5 = moderate headaches, and 10 = worst possible headache).

‡ Value presented for respondents who reported headache (n = 32).

^§^ Surgeons were asked to rank their average level of neck or back pain/discomfort on a scale of 0–10 (0 = no pain, 5 = moderate pain, and 10 = worst possible pain).

**Abbreviations**: HUD = heads-up display; IQR = interquartile range; SD = standard deviation.

### Device preference

Forty-six (69%) surgeons reported that they preferred HUD to microscope, and 57 (85%) selected that they would recommend it to their peers. The main reasons reported for HUD preference were visualization (13; 28%), improved posture/ergonomics (11; 24%), and education (10; 22%). Additional reasons for preference related to ergonomics were comfort (7; 15%) and reduced fatigue (6; 13%) (**Table 3**). Of 21 (31%) surgeons who preferred conventional microscope, visibility of certain areas (10; 48%), ease-of-use (5; 24%), and resolution (3; 14%) were the top reasons provided.

**Table 3:** Reasons for device preference.

Preference for Heads-up Display	*n* = 46*	Preference for Conventional Microscope	*n* = 21*^,^^†^
Visualization	13	Visibility of certain areas	10
Improved posture/ergonomics	11	Ease of use	5
Education	10	Resolution	3
Magnification	8	Minimal benefit to HUD	1
Comfort	7	Posture	1
Reduced fatigue	6	Reduced fatigue	1
Future potential	4	Comfort	1
Better overall	2		
Better performance	1		
Patient benefit	1		

* Responses were not mutually exclusive.

† One individual selected HUD but stated “Currently case by case. I select both.”

**Abbreviations:** HUD = heads-up display.

### Presence of MSDs and concerns for long-term impacts

Forty-six (69%) participants reported some degree of headache and/or neck or back pain/discomfort, and 27 (40%) participants reported that they had received treatment (e.g., physiotherapy or acupuncture) to manage their MSDs. Trouble, defined by the NMQ as ache, pain, discomfort, or numbness, was most often reported in the lower back (35; 52%), followed by the neck (32; 48%). When asked about the effects of long-term impacts, of the 42 (63%) respondents that expressed experiencing trouble, some reported changes to clinical practice as a result: five (12% of applicable) decreased a specific type of surgery, three (7% of applicable) decreased clinic hours, and one (2% of applicable) decreased time in surgery. Thirty surgeons (45%) believed that the use of conventional microscope over the course of their career has had a negative impact on their health.

### Multivariate analysis

Multivariable logistic regression analysis was performed to explore which variables were likely to be predictors of improvement in pain and/or discomfort while operating. Stepwise selection for the best model resulted in three variables being retained: years of practicing ophthalmology, years of experience using HUD, and proportion of surgeries performed with HUD. The multivariate analysis indicated that an improvement in pain and discomfort was significantly correlated with the proportion of surgeries performed with HUD (*P* = 0.018, **Table 4**). The model also suggested years of practicing ophthalmology and years of experience using HUD were important predictive variables; however, these did not reach statistical significance (*P* = 0.092 and *P* = 0.353, respectively).

**Table 4:** Multivariable logistic regression analysis for predictors of improvement in pain/discomfort in the operating room since introducing heads-up display.

Variables	Estimate (SE)	Z	*P*-value
Intercept	-2.073 (1.947)	-1.065	0.287
Years of experience with HUD	0.571 (0.615)	0.928	0.353
Years practicing ophthalmology	0.147 (0.087)	1.684	0.092
Interaction effect: Years of experience with HUD x Years practicing ophthalmology	-0.043 (0.026)	-1.682	0.093
Proportion of surgeries performed with HUD	2.307 (0.973)	2.372	0.018

**Abbreviations**: HUD = heads-up display; SE = standard error.

## Discussion

This observational, cross-sectional study compared ergonomic outcomes with the use of HUD versus conventional ocular microscopes in the OR and collected novel data on the presence of MSDs and expected long-term impacts in ophthalmic surgeons in Japan. Overall, the results of the current study from Japan were in line with previous literature from other countries demonstrating that many ophthalmologists are experiencing occupational MSDs [2–5]. As seen in a similar survey administered to US ophthalmologists [5], a high proportion of respondents in this sample reported improvements with pain/discomfort during operation and overall trouble with the introduction of HUD, with the majority indicating a preference for HUD to conventional microscope. Moreover, in addition to ergonomic benefits, a high proportion also reported HUD benefits for education (82%) and visualization (58%).

Notably, a much greater proportion of surgeons reported upright head posture with HUD (72%) than with conventional microscope (34%); similarly, a greater proportion reported sitting upright (61% vs. 33%) or slightly leaned back (24% vs. 1%) with HUD than with conventional microscope, respectively. As previously mentioned, this sustained forward head posture (FHP) caused by the use of a microscope is a key contributor to neck-related MSDs [7]. It has been shown that for every 2.5 cm that the head is held forward in poor posture, an additional 4.5 kg of weight is felt on the cervical spine [16]. Since the average head weighs between 4.5 and 5.5 kg, this means that even 2.5 cm of FHP may double the load on the cervical spine [16]. A systematic review of the relationship between FHP and neck pain revealed that neck pain measures in adults were significantly correlated with FHP, with those experiencing neck pain showing increased FHP compared to asymptomatic individuals [17]. Additionally, FHP has been associated with tension headaches, caused by abnormal muscle tone around the skull and cervical spine [18]. Prolonged posture with excessive neck bending has also been associated with upper cross syndrome, leading to muscles of the shoulders, neck, and chest to be out of balance [19]. By encouraging upright posture, the use of HUD in the OR may mitigate these undesirable musculoskeletal outcomes. It is important to acknowledge that self-reported head position data is not as reliable as objective measurements if self-awareness of posture is inaccurate. Future studies should consider utilizing more objective methods of measurement to validate these findings, such as semi-quantitative tools administered by a secondary observer [20, 21].

With the exception of restrictions to surgery types, concerns for long-term impacts of MSDs were lower than what has been shown in the literature [1, 4]. In the present study, no respondents reported that they stopped operating, considered switching careers, or considered retiring early due to trouble, whereas a survey of MSDs in US ophthalmologists found that 14% reported early retirement and 2% reported a career change due to musculoskeletal pain [4]. Nonetheless, the present results indicate MSD-associated productivity loss, with 12%, 7%, and 2% of applicable surgeons reporting a decrease in performing a specific type of surgery, decreased clinic hours, or decreased time performing surgery, respectively. These findings were in accordance with a meta-analysis of work-related MSDs amongst surgeons which reported that 12% of physicians with work-related MSDs require a leave of absence, practice restriction/modification of surgical practices, or early retirement [1].

Compared to outcomes reported by Weinstock et al. [5] in their survey of US ophthalmologists, Japanese surgeons seemed more conservative in their level of agreement for the negative effect of microscope (45% vs. 61% for Japan and US surgeons, respectively), improvement of pain/discomfort with HUD (72% vs. 82%) and improvement in trouble with HUD (63% vs. 75%), despite similar proportions between Japan and US cohorts for baseline pain levels reported on NMQ. Possible explanations for this discrepancy may be the higher response rate of the current study (94% vs. 42%), which may indicate a more representative sample of the real world, potential differences in physical characteristics between Japanese and American respondents (e.g., average height was 171.76 cm [Japanese] vs. 178.55 cm [American], average age was 51.15 years vs. 45.55 years [Wilcoxon signed rank test *P* < 0.001 and *P = 0*.*001*, respectively, Supporting Information (S2 File)]), or potential cultural differences between American and Japanese surgeons when reporting MSDs or perceiving benefits of HUD. Despite the slightly lower level of agreement for benefits, similar proportions of Japanese and US surgeons reported preference for HUD (69% and 69%, respectively) and HUD recommendation to their peers (85% and 86%, respectively).

In the US study, surgeons were distinctly split by specialty into anterior-segment and posterior-segment subgroups, which enabled an evaluation of any significant differences between specialties [5]. Differences between these subgroups in the US were observed in average case length, estimated annual case volume, and self-reported average baseline neck or back pain, with lower median baseline pain levels reported in posterior-segment surgeons [5]. Since Japanese surgeons typically specialize in multiple surgery types, the Japanese cohort was divided based on the majority of performed surgeries. It was found that the cataract-specialty surgery subgroup had the lowest average case length but the highest annual case volume, while the anterior surgery subgroup (i.e., sum of anterior eye surgeries [glaucoma and corneal surgery] greater than the sum of posterior eye surgeries [retinal surgery]) reported the longest average case time and lowest annual case volume. Proportions of surgeons experiencing baseline neck pain were higher in the anterior and posterior groups than the cataract-specialty group. In contrast to the US study, the highest baseline neck or back pain levels were reported by the posterior surgery group.

One of limitations of the present study is that respondents were recruited from a device-install list, which may reduce the reach and generalizability of results to all ophthalmologists. As such, there may be an existing bias for the belief in benefits of HUD or presence of MSDs, leading the surgeons to purchase HUD as a means of mitigating pain. In an attempt to limit this bias, surgeons were required to have previous experience with both devices, with questions specifying that surgeons should directly compare devices by thinking back to when they only used the microscope. Moreover, some of the respondents only used HUD in low proportions. Nonetheless, the very high response rate in the current study created a well-rounded sample, reducing the chance of bias that respondents were only those who have a strong preference for HUD. In fact, the present sample size was similar [5] or higher [11–13] than much of the existing literature in this space. Another limitation is the survey-based nature of the study, which creates potential for recall bias. While this was minimized by limiting many questions to the previous 12-month period, the questionnaire-based design was necessary to allow for responses based on real-world conditions and experiences from a larger sample, as opposed to a controlled testing environment, thereby supporting external validity.

In conclusion, this study presents novel information about the prevalence of MSDs in Japanese ophthalmologists and how they believe this could impact their career, contributing knowledge to an important area of focus [6, 22]. Specifically, this study built on existing literature by comparing reported ergonomic benefits of HUD with conventional ocular microscope in a novel population, Japanese ophthalmologists. Similar to previous findings, the majority of respondents identified ergonomic benefits of HUD and reported a preference for this technology. Advancing ergonomics in the OR is critical to promote well-being and productivity in surgeons. Future studies using objective measures such as a randomized prospective design or electromyogram are warranted to provide unbiased ergonomic information and outcomes of HUD.

## Supporting information

S1 File Questionnaire. (DOCX)

S2 File Comparison of Baseline characteristics between US and Japan studies. (DOCX)

S3 File Minimal data set underlying results. (XLSX)
