# Supplementary material for: Ergonomic benefit using heads-up display compared to conventional surgical microscope in Japanese ophthalmologists
Source: PLoS One. 2024 May 22;19(5):e0297461. doi: 10.1371/journal.pone.0297461 (PMC11111003; doi:10.1371/journal.pone.0297461)
Supplement: S1 File — (DOCX) [file pone.0297461.s001.docx]

# Supporting Information File 1: Questionnaire

**Part 1: Collection of Surgeon and Procedure Demographics and Characteristics**

| **Question** | **Response Type** |
| --- | --- |
| 1a) Position | MC & OE (resident, fellow, attending, other [please specify]) |
| 1b) Specialty | MC (retinal, cataract, glaucoma, corneal specialist or none of the above) |
| 1c) Please estimate the distribution of your procedures that would be described as the following, ensuring the total sum of the distribution is 100% | Retinal surgeries only; cataract surgeries only; glaucoma surgeries only; corneal surgeries only; combination cataract and retinal; combination cataract and glaucoma; combination retinal and glaucoma; combination retinal, cataract, and glaucoma; combination corneal and others (__% entry for all) |
| 1d) Number of years practicing ophthalmology, 1e) Age | Number select (years) |
| 1f) Sex | MC (M/F/refuse to answer) |
| 1f) Height (cm), 1g) Weight (kg) | Number select |
| 1i) Do you work at a teaching facility? | Yes/No |
| 1j) If Yes, please estimate the proportion of time you occupy the assistant versus the surgeon chair. | Number select (% assistant, % primary surgeon) |
| 2a) Do you suffer from chronic neck or back pain/discomfort? “Chronic pain/discomfort’ can be defined as either persistent daily or occasional but recurring. | Yes/No |
| 2b) If yes, for how long have you suffered from chronic neck or back pain/ discomfort? | Number select |
| 3) Please rate your average headache severity on a scale of 0-10. Select 0 if you do not experience headaches. | Number select |
| 4) Please rate your average level of neck or back pain/discomfort on a scale of 0-10. Select 0 if you do not experience neck or back pain. | Number select |
| 5) Do you have a history of injury (eg, a sports injury or car accident) or surgery to the neck or back? If yes, when did it occur? | Yes/No  Select (injury, surgery, other) Month/Year |
| 6) Have you obtained medical treatment by a doctor, physiotherapy, or acupuncture for services for musculoskeletal pain? | Yes/No |
| 7a) Have you missed any time at work, including operating room time, due to pain? | Yes/No |
| 7b) If yes, approximately how much work have you missed due to pain in the past 12 months? | Select (1-7 days, 8-30 days, more than 30 days) |
| 8) In your opinion, which procedure types result in the most physical strain, for which you would desire improved ergonomics? | OE |
| 9) Which of the following best describes the position of the heads-up display relative to your head when you are operating? | 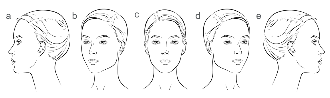 |
| 10) Which of the following best resembles your posture when using a conventional microscope? | 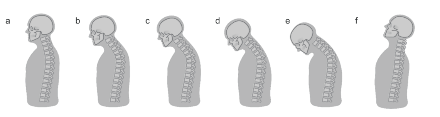 |
| 11) Which of the following best resembles your posture when using heads-up display? | 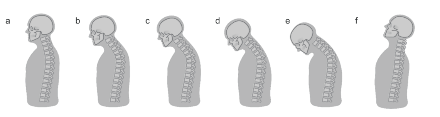 |
| 12) Which of the following best resembles the angle of your back when using a conventional microscope? | 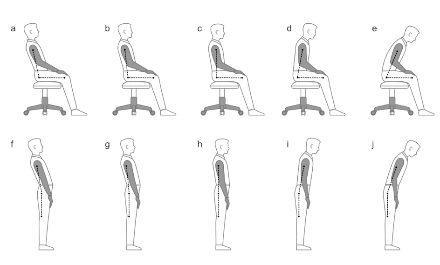 |
| 13) Which of the following best resembles the angle of your back when using heads-up display? | 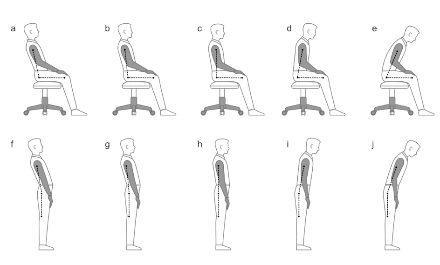 |
| 14) When did you personally start using a heads-up display for surgeries? | MC (month/year) |
| 15) Which heads-up display system have you used most often? | MC & OE (NGENUITY, TrueVision, ARTEVO 800, other) |
| 16) Please estimate the total number of procedures you have completed with this technology in the past 12 months: | Number select |
| 17) When was your most recent procedure with a conventional microscope? | MC (This week, last week, 2-4 weeks ago, 2-6 months ago, 6-12 months ago, >1 year ago) |
| 18) When was your most recent procedure with a heads-up display? | MC (This week, last week, 2-4 weeks ago, 2-6 months ago, 6-12 months ago, >1 year ago) |
| 19) Which factors determine the use of the heads-up display at your facility? Please select all that apply. | Select from list (Educational purposes; Length of procedure; Procedural type; Surgeon seniority/tenure; Ergonomic requirements; Visualization requirements; Availability/ advanced booking only; I do not know; Other (please specify)) |
| 20) How long is a typical operating session for you (ie, the window of time you block/ the number of consecutive hours of surgery you typically perform on a surgical day)? | Number select (hours, minutes) |
| 21) Please estimate the average time it takes you to complete the surgery you perform most frequently: | Number select (hours, minutes) |
| 22a) Please estimate the total number of cases you complete in a typical day and annually. | Number select |
| 22b) Please estimate the proportion of your cases completed with heads-up display. | Percent entry |
| 23a) How many rooms do you operate out of for ophthalmology cases at your facility? | Number select |
| 23b) How many of these rooms have a heads-up display? | Number select |
| 24) Please select the operating position used in the majority of your cases. | Select (Superior (in >90% of cases); Temporal (in >90% of cases); Mixed distribution) |
| 25) With which brand(s) of microscope(s) is the heads-up display integrated? | Select (from various) |
| 26) Please describe the type of microscope. | Floor-mounted/Ceiling mounted |

MC=Multiple choice; OE=Open ended.

**Part 2: Nordic Musculoskeletal Questionnaire**

See Kuorinka et al, (1987)^15^ Figure 1, Panel 3 (Page 3).

| **Question** | **Response Type** |
| --- | --- |
| 1) Have you at any time during the last 12 months had trouble (ache, pain, discomfort, numbness) in: a) Neck b) Upper Back c) Lower Back d) Shoulders (Right/Left) | Yes/No |
| 2) Have you at any time during the last 12 months been prevented from doing your normal work (at home or away from home) because of the trouble? a) Neck b) Upper Back c) Lower Back d) Shoulders (Right/Left) | Yes/No |
| 3) Have you had trouble at any time during the last 7 days? a) Neck b) Upper Back c) Lower Back d) Shoulders (Right/Left) | Yes/No |

**Part 3:** The Impact of Musculoskeletal Disorders on Surgery

| **Question** | **Response Type** |
| --- | --- |
| 1) Has your trouble (ache, pain, discomfort, numbness) caused you to do any of the following? If you have ***not*** experienced musculoskeletal pain or discomfort in your career, please select “not applicable”: |  |
| 1. Decrease your clinic hours   If yes, by what proportion have you decreased your clinic hours? | Yes/No/N/A  Range 1-100% |
| 1. Decrease your time spent performing surgery   If yes, by what proportion have you decreased your time in surgery? | Yes/No/N/A  Range 1-100% |
| 1. Decrease a specific type of surgery   If yes, which types of procedures have you stopped performing? | Yes/No/N/A  OE |
| 1. Stop operating entirely |  |
| 1. Consider switching careers |  |
| 1. Consider retiring early   If yes: What age did you initially think you would retire at? What age do you now plan to retire at?  Do you think you could operate for longer if your pain/discomfort troubles were alleviated? | Number select (years of age)  OE |
| 2) In the past 12 months have you altered your surgical plan to accommodate any pain/discomfort (eg, altered the types of tools or equipment you work with, or altered the surgical technique you use to avoid discomfort or pain)?  If yes, please describe what strategies have you used to mitigate or prevent any pain/discomfort? | Yes/No/N/A  OE |
| 3) Are you concerned that issues with pain/discomfort may impact your ability to perform future surgeries? | Yes/No/N/A |
| 4) Do you have any other concerns about the potential long-term impacts from occupational ergonomic stress? | Yes/No/N/A |

N/A=Not Applicable; OE=Open ended.

**Part 4: Custom questions** **comparing heads-up displays and microscope**

| **Question** | **Response Type** |
| --- | --- |
| 1) Do you feel using a conventional microscope over your career has had a negative impact on your health?  If yes, how? | Yes/No  OE |
| 2) Have you noticed improvements with eyestrain since using the heads-up display in the operating room, compared with conventional microscope? | Yes/No/N/A |
| 3) Do you currently feel less pain/discomfort when you operate heads up compared with conventional microscope? | Yes/No/N/A |
| 4) Thinking back to a time when you ONLY used conventional microscope, has your trouble (ache, pain, discomfort, numbness) today improved with the use of heads-up display? | Yes/No/N/A |
| 5) COMPARED TO CONVENTIONAL MICROSCOPE, the use of a heads-up visualization system in the operating room  a) has reduced the severity of pain and discomfort  b) has reduced the frequency of pain and discomfort  c) has improved my posture  d) has improved my overall comfort during surgery  e) has improved my mental performance (eg, ability to focus, mental clarity)  f) has improved my physical performance (eg, fatigue, stamina, mobility)  g) has improved my confidence in my ability to perform complex procedures  h) allows me to better visualize the areas and angles required for the procedure  i) allows me to operate more comfortably under higher magnification  j) has reduced the severity of headaches  k) has reduced the frequency of headaches  l) allows for better education of surgical techniques | MC (strongly disagree, disagree, neutral, agree, strongly agree) |
| 6) All things considered, do you prefer the heads-up display or conventional microscope? | MC |
| 6a) What is the main reason for this preference? | OE |
| 7) Would you recommend heads-up visualization to your peers? | Yes/No |

MC=Multiple choice; N/A=Not Applicable; OE=Open ended
