# Supplementary material for: Ergonomic benefit using heads-up display compared to conventional surgical microscope in Japanese ophthalmologists
Source: PLoS One. 2024 May 22;19(5):e0297461. doi: 10.1371/journal.pone.0297461 (PMC11111003; doi:10.1371/journal.pone.0297461)
Supplement: S2 File — (DOCX) [file pone.0297461.s002.docx]

# Supporting Information File 2: Comparison of Baseline Characteristics between US and Japan Studies

| **Characteristic** | **US**  **(n=64)** | **Japan**  **(n=67)** | **FDR Corrected  P-Value*** |
| --- | --- | --- | --- |
| **Sex**^†^  Female (n, %)  Male (n, %)  Refused to answer | 8 (12.5%)  55 (85.9%)  1 (1.6%) | 3 (4%)  64 (96%)  0 (0%) | 0.190 |
| **Height, cm (mean, SD)** | 178.55 (11.94) | 171.76 (5.99) | **< 0.001** |
| **Weight, kg (mean, SD)** | 78.71 (11.38) | 71.37 (10.53) | **< 0.001** |
| **Age (mean, SD)** | 45.55 (9.79) | 51.15 (8.67) | **0.001** |
| **Years practicing ophthalmology (mean, SD)** | 14.88 (9.44) | 25.00 (8.86) | **< 0.001** |
| **Years using heads up display (mean, SD)** | 2.07 (1.78) | 2.73 (1.69) | **< 0.001** |
| **Operating position**^†^  Temporal in >90% of cases (n, %)  Superior in >90% of cases (n, %)  Mixed distribution (n, %) | 21 (32.8%)  40 (62.5%)  3 (4.7%) | 6 (9.0%)  52 (77.6%)  9 (13.4%) | 0.363 |
| **Type of Microscope**  Floor Mounted (n, %)  Ceiling Mounted (n, %) | 55 (85.9%)  9 (14.1%) | 54 (80.6%)  13 (19.4%) | 0.398 |
| **Average case length (mean, SD)**^‡^ | 33.91 (22.29) | 32.22 (29.04)^§^ | **< 0.001** |
| **Estimated annual case volume (mean, SD)** | 490.70 (467.43) | 730.87 (568.13) | **0.010** |
| **Estimated proportion of cases completed with heads up display (n surgeons, %)**  50% and Under  Over 50% | 31 (49.2)  32 (50.8) | 36 (53.7%)  31 (46.3%) | 0.863 |
| **Headache severity^**^ (median, IQR)** | **1.00 (0 – 3.00)** | **0.00 (0 – 2.00)** | 0.057 |
| **Level of neck or back pain/discomfort**^††^ **(median, IQR)** | 2.00 (1.00 – 3.00) | 1.00 (0 – 4.00) | 0.270 |
| **Nordic Musculoskeletal Questionnaire:**  No pain (n, %) | 20 (31.2%) | 22 (32.8%) | 1.00 |
| **Nordic Musculoskeletal Questionnaire:**  Neck (n, %)  Upper Back (n, %)  Lower Back (n, %)  Shoulder (right) (n, %)  Shoulder (left) (n, %) | 31 (48.4%)  20 (31.2%)  26 (40.6%)  11 (17.2%)  11 (17.2%) | 32 (47.8%)  21 (31.3%)  35 (52.2%)  19 (28.4%)  21 (31.3%) | 1.00  0.383  1.00  0.372  0.165 |

* P-value compared United States data (originally published in Weinstock et al., 2021) with Japan data. Wilcoxon signed rank test was used to compare absolute means. Test of proportion was used to compare mean differences in proportions.

† Assessed male category versus any other category. Assessed superior category versus any other category.

‡ In the US, surgeons specialize as either anterior- or posterior-segment surgeons, while in Japan, surgeons are multi-specialty, and may perform combination surgeries. Question was phrased differently for US questionnaire (“Please estimate the time it takes you to complete an average case (ie, a surgery)”) compared to Japan questionnaire (“Please estimate the average time it takes you to complete the surgery you perform most frequently”).

^§^ Value presented after removal of outliers (n=3) who reported abnormally long durations for one procedure (12, 8, and 6 hours).

**Surgeons were asked to rank their average headache severity on a scale of 0-10 (0 = no headaches, 5 = moderate headaches, and 10 = worst possible headache).

^††^Surgeons were asked to rank their average level of neck or back pain/discomfort on a scale of 0-10 (0 = no pain, 5 = moderate pain, and 10 = worst possible pain).
